# Youth-Onset Type 2 Diabetes Before and After COVID-19 Pandemic-Related Public Health Restrictions: Trends in Incidence, Severity, and Remission

**DOI:** 10.3390/jcm14227995

**Published:** 2025-11-11

**Authors:** Jody Beth Grundman, Elizabeth Estrada, Rachel Longendyke, Stephanie T. Chung

**Affiliations:** 1 Division of Endocrinology, Children’s National Hospital, Washington, DC 20010, USA; 2 Section of Pediatric Diabetes, Obesity, and Metabolism, National Institute of Diabetes and Digestive and Kidney Diseases, Bethesda, MD 20892, USA; stephanie.chung@nih.gov

**Keywords:** pediatric type 2 diabetes, COVID-19, remission, lifestyle habits, health disparities

## Abstract

**Background/Objectives**: Youth-onset type 2 diabetes (Y-T2D) incidence and severity rose during the COVID-19 pandemic, particularly during periods of widespread public health restrictions—including, but not limited to, virtual learning, stay-at-home orders, closure of recreational facilities, and limitations on in-person healthcare access. This study assessed incidence, severity, and remission rates of Y-T2D following the return to in-person education, focusing on cases diagnosed while such restrictions were in place. **Methods**: A retrospective chart review was conducted at a pediatric tertiary care center (2018–2024) to identify new Y-T2D diagnoses. We compared incidence rates, disease severity at diagnosis, and remission outcomes before and after the period of comprehensive public health restrictions, defined locally as March 2020–August 2021, during which virtual learning was implemented. **Results**: Incidence declined from 13.2 to 6.3 cases/month after the major restrictions were lifted. Youth diagnosed after the restrictions period had lower rates of diabetic ketoacidosis (7.1% vs. 20.9%, *p* < 0.001) and severe hyperglycemia (HbA1c 9.1 ± 2.5% vs. 10.1 ± 2.3%, *p* < 0.001). Among those diagnosed during the restriction period, 11.1% achieved remission within three years. Remission was associated with lower baseline HbA1c (OR = 9.52, 95% CI: 2.2–41.7, *p* = 0.003), metformin use (OR = 7.0, CI: 1.9–26.3, *p* = 0.004), GLP-1 receptor agonist use (OR = 5.8, CI: 1.3–24.4, *p* = 0.018), and lower likelihood of insulin therapy (OR = 19.5, CI: 2.3–166.7, *p* = 0.007). **Conclusions**: The reduction in Y-T2D cases after the lifting of pandemic-related restrictions highlights the impact of pandemic-related environmental changes. Low remission rates—especially among underserved youth—underscore the urgency of early screening, prompt intervention, and equitable access to pediatric diabetes care, and highlight the need to consider the metabolic health impacts of future prolonged public health measures.

## 1. Introduction

The prevalence and severity of presentation of youth-onset type 2 diabetes (Y-T2D) increased dramatically during the COVID-19 pandemic [1,2,3,4,5]. The reasons for the rapid and steep increase in rates were multi-factorial and likely related to structural, socio-economic, and behavioral factors. Notably, pandemic-imposed public health restrictions—including virtual learning, closure of recreational facilities, stay-at-home orders, restrictions on social gatherings, and reduced in-person healthcare access —contributed to new cases of Y-T2D [6].

Although the most intense restriction period, which included full virtual learning, was relatively short (March–August 2020 in our region), the impact on youth metabolic health was profound. Virtual learning coincided with an approximately five-fold increase in Y-T2D incidence, with up to 50% of patients presenting with severe diabetic ketoacidosis [6]. Overall, COVID-19 infection rates at diagnosis were low (2.5%) and BMI trends remained stable, suggesting that factors unique to the socio-behavioral environment during this restriction period may be causally linked to Y-T2D onset [6]. The shift to virtual learning during the COVID-19 pandemic served as a proxy for a broad set of environmental and behavioral disruptions, including reduced physical activity, increased screen time, social isolation, and altered eating and sleep patterns. It also overlapped with policy-driven limitations on sports participation, closure of parks and playgrounds, and delays in routine preventive healthcare. The return to in-person education allowed for the re-establishment of structure, routine physical activity, peer engagement, and access to school-based resources. These time periods offer a natural experiment to evaluate the impact of social context and multi-layered public health restrictions on Y-T2D presentation and outcomes.

With the return to in-person learning, incident rates of Y-T2D declined. We postulated that this trend could be related to resumption of habitual lifestyle, dietary habits, and decreased emotional distress. However, causal relationships were out of scope for the previous analysis due to a lack of patient-level data on exercise, sleep, diet, and psychological stress [6]. The COVID-19 pandemic restrictions leading to virtual learning could be viewed as a modern era social experiment with an abrupt onset and off-set [7,8]. Therefore, we systematically collated data to longitudinally assess the epidemiology of Y-T2D in our diabetes center. The aim of this follow-up study was to compare changes in incidence rates and presentation severity in Y-T2D diagnosed in the two-year period after the restrictions period with those during the pandemic restrictions period, and to determine remission rates and characteristics of Y-T2D diagnosed during the restrictions period. This study extends previously published data by incorporating post-restriction outcomes through 2024 and introducing remission analysis, providing one of the longest continuous Y-T2D follow-ups reported to date.

## 2. Materials and Methods

The restriction period was defined as March 2020 through August 2021, corresponding to the duration of virtual learning and local public health mandates in the District of Columbia. Temporal boundaries were chosen to align with the period of widespread school closures and virtual learning in the Washington, DC metropolitan area, acknowledging that local reopening policies varied. All references to this timeframe have been standardized. This period corresponds with local policy changes, including the closure of schools and transition to virtual learning, cancellation of extracurricular sports, closure of public recreational facilities, and limits on in-person outpatient visits. The post-restrictions period began with the resumption of in-person education and the gradual lifting of these measures. Pre-pandemic incidence data from 2018 to March 2020 were referenced only for historical context. We conducted a retrospective cross-sectional review of medical records to identify all cases of newly diagnosed Y-T2D evaluated at Children’s National Hospital, a tertiary pediatric referral center in Washington, DC, USA, from 11 March 2020 to 10 March 2024. Results demonstrating the rise in incidence rates during the first 2 years of the COVID-19 pandemic during and after the restrictions period (11 March 2020 to 10 March 2022) have been published [6]. This range reflects the Washington, DC, USA metropolitan region’s period of formal public-health restrictions and school closures; although adherence varied by district, this timeframe encompassed the majority of virtual-learning months in our catchment area.

Data were collected for an additional two years after the start of the COVID-19 pandemic (11 March 2022–10 March 2024). The diabetes program systematically records comprehensive education logs for all individuals younger than 21 years receiving instruction at the time of diabetes onset. These education records served as the source to capture every new diagnosis of Y-T2D within the four-year study window. Participants were included when meeting ADA diagnostic thresholds for diabetes—either fasting glucose ≥ 126 mg/dL, A1c ≥ 6.5%, or random glucose ≥ 200 mg/dL accompanied by hyperglycemic symptoms [9]—while demonstrating no more than one positive pancreatic autoantibody [1], and having obesity, defined as a BMI Z-score greater than 1.64 [10]. Measurement of pancreatic autoantibodies (glutamic acid decarboxylase 65 kDa isoform (GAD65), islet antigen 2 (IA2), insulin, zinc transporter 8, and islet cell antibody) was performed for hospitalized patients and, for outpatients, when ordered at the clinician’s discretion (n = 295). For individuals lacking antibody data (n = 22) or presenting with only one positive antibody (n = 2), a pediatric endocrinologist reviewed the medical record to confirm that clinical features—such as family history, insulin needs, and longitudinal weight trends—were compatible with youth-onset type 2 diabetes [1]. The diabetes education logs systematically track demographic and clinical data but were not designed to capture behavioral variables such as diet, sleep, exercise, or psychosocial stress. These data were not routinely recorded in a structured format during the pandemic, limiting causal inferences about lifestyle mediators.

During all study years, patients who arrived at their primary provider well-hydrated, without notable weight loss, and with stable laboratory findings—glucose < 250 mg/dL, A1c < 10%, and trace or negative urinary ketones—were considered suitable for same-day outpatient endocrine assessment. Routine laboratory assessments for new-onset T2D included electrolytes, anion gap, creatinine, and blood urea nitrogen (BUN). Venous pH was obtained in a youth requiring hospital admission. Inpatient admission criteria include: severe hyperglycemia (serum glucose > 300 mg/dL (16.7 mmol/mL) after saline bolus or HbA1c > 11% (96.7 mmol/mol)), moderate to large urine ketones, dehydration, weight loss, challenging social circumstances, or an inability to coordinate urgent outpatient follow-up [1]. The clinical thresholds guiding hospital admission for presumed new-onset youth-onset type 2 diabetes were consistent across the study years. Access to urgent outpatient appointments was constant over time and did not affect how many patients ultimately required hospitalization.

Demographic and laboratory data at diagnosis (serum bicarbonate, venous pH, HbA1c, COVID-19 status), weight, body mass index (BMI), and BMI Z-scores ±30 days from diagnosis were recorded. Classification of diabetic ketoacidosis and hyperosmolar DKA followed the standards established by the International Society for Pediatric and Adolescent Diabetes [11]. DKA was defined by plasma glucose > 200 mg/dL (11.1 mmol/L), venous pH < 7.3 or serum bicarbonate < 15 mmol/L, and ketonuria or ketonemia [11]. DKA severity was classified as either mild (pH < 7.3 or bicarbonate < 15 mmol/L), moderate (pH < 7.2 or bicarbonate < 10 mmol/L), or severe (pH < 7.1 or bicarbonate < 5 mmol/L) [11]. Hyperglycemic hyperosmolar syndrome was defined by plasma glucose > 600 mg/dL (33.3 mmol/L), effective serum osmolality > 330 mOsm/kg, and pH > 7.25 or serum bicarbonate > 15 mmol/L whereas hyperglycemic hyperosmolar DKA was defined by effective serum osmolality > 330 mOsm/kg, plasma glucose > 600 mg/dL (33.3 mmol/L), and pH ≤ 7.25 or bicarbonate ≤ 15 mmol/L [12]. When laboratory data was incomplete, classification of youth-onset T2D was made by consensus among endocrinologists. Obesity was categorized by class (Class 1 obesity: ≥95th percentile; Class 2 obesity: ≥120% of the 95th percentile or BMI ≥ 35 kg/m^2^, whichever is lower; Class 3 obesity: ≥140% of the 95th percentile or ≥40 kg/m^2^, whichever is lower) [13].

Y-T2D remission was defined by HbA1c < 6.5% at 3 months after stopping glucose-lowering medication [14]. Review of the EMR was used to capture the following: number of follow-up visits, T2D duration, medication regimen (Metformin/Riomet, Glucagon-Like-Peptide-1 (GLP-1), Sodium-Glucose Costransporter 2 (SGLT-2), thiazolidinedione (TZD), insulin), HbA1c, BMI, weight, and obesity class. De-identified data and analytic code are available upon reasonable request.

### Statistical Analyses

Demographic data was compared according to those who were not in remission on glucose-lowering medication, not in remission and not on glucose-lowering medication, in remission, and those with no follow-up visit data. Changes in HbA1c, weight, and BMI were evaluated for those not in remission on glucose-lowering medication, not in remission and not on glucose-lowering medication, and those in remission. Differences in changes in HbA1c, weight, and BMI were compared among those who achieved remission and those who did not.

Statistical analysis was performed using Statistical Package for the Social Sciences (SPSS version 30) [15]. Chi-square tests, independent samples *t*-tests, and ANOVA were used to evaluate between-group differences for baseline variables during the time periods and follow-up variables after Y-T2D diagnosis. Binomial logistic regression was used to assess the effects of age, biological sex, race/ethnicity, insurance type, baseline HbA1c, weight, BMI, changes in HbA1c, weight, and BMI, and use of glucose-lowering medications on remission status. Pairwise deletion was used for missing data (HbA1c n = 4; venous pH n = 100; weight n = 1; BMI n = 4). Data are mean ± SD or number (percent) unless otherwise stated. A *p*-value of <0.05 was used to define statistical significance.

Although the number of youth diagnosed post-restrictions period (n = 154) was smaller than during the restrictions period (n = 235), power calculations indicated sufficient sample size to detect a 10% absolute difference in remission rates (α = 0.05, β = 0.80). Welch’s *t*-tests and chi-square tests with Yates correction were used to account for unequal group sizes. To further contextualize findings, effect sizes (Hedges’ g for continuous variables and Cramér’s V for categorical variables) were calculated and reported. Because remission occurred in a small subgroup, logistic models were limited to a small number of predictors to avoid overfitting. This study was designed as a descriptive, retrospective chart review intended to characterize temporal associations rather than infer causality.

## 3. Results

**Figure 1:**
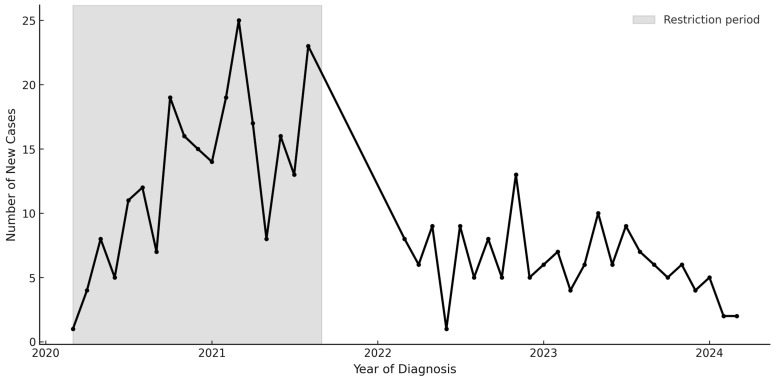
New Diagnoses of youth-onset type 2 diabetes from 2018–2024.

154 youth were diagnosed with T2D in the 2-year post-restrictions period (Table 1); most youth were publicly insured, and over 80% identified as non-Hispanic Black (NHB) or Latinx. Incident cases of T2D decreased from 13.2 cases/month during the restrictions period to 6.3 cases/month post-restrictions, compared with a verified pre-pandemic baseline of 3.9 cases per month. Incident cases post-restrictions remained elevated compared to pre-pandemic Y-T2D incidence (3.9 cases/month) (Figure 1). A greater proportion of those who identified as Latinx (18.7% vs. 29.9%, *p* = 0.003) and were on private health insurance (16.2% vs. 29.9%, *p* < 0.001) were diagnosed with Y-T2D post-restrictions period compared to during public health restrictions. The frequency of DKA and severe hyperglycemia differed markedly across periods. During restrictions, 20.9% of new cases presented with DKA compared with 7.1% after restrictions were lifted (*p* < 0.001). Severe DKA (pH < 7.1 or bicarbonate < 5 mmol/L) occurred in 8.5% of restriction-period cases but only 2.6% post-restrictions. Median random blood glucose, serum bicarbonate, and HbA1c likewise reflected greater metabolic decompensation during restrictions.

Fifty-four percent (n = 126) of Y-T2D diagnosed during the restrictions period followed up for continued Y-T2D outpatient clinical care. Youth seen in outpatient clinic, compared to youth lost to follow up, were significantly older (age 15.2 ± 2.2 vs. 13.8 ± 2.0, *p* < 0.001) and had higher HbA1c at diagnosis (HbA1c 10.1 ± 5.2% vs. 7.5 ± 2.7%, *p* < 0.001), but did not differ significantly with respect to weight (weight 111.1 ± 26.6 kg vs. 106.2 ± 23.7 kg, *p* = 0.143) and BMI at diagnosis (BMI follow up 37.7 ± 7.5 kg/m^2^ vs. 37.1 ± 7.4 kg/m^2^ no follow up, *p* = 0.538) when compared to those that did not follow up outpatient.

Patients with follow-up data had a median of 3.5 follow-up visits (IQR 2,5) and 14 months (IQR 9,17) of follow-up time in the 2-year follow-up observation period. Among the newly diagnosed, 11.1% (n = 26) of youth diagnosed with Y-T2D during public health restrictions achieved remission (Table 2). Race/ethnicity differed significantly by remission status (*p* = 0.047). There were no significant differences with respect to insurance status or biological sex (*p* = 0.623, *p* = 0.669, respectively). To assess the impact of loss to follow-up, we conducted a sensitivity analysis. If all patients lost to follow-up achieved remission, the remission rate would rise to 26.4%. In contrast, if none of the patients lost to follow-up achieved remission, the remission rate remained at 11.1%. These boundary estimates suggest our primary remission finding may underestimate the true rate. Descriptive comparisons also show that those lost to follow-up were younger and had lower HbA1c, both of which were associated with a higher likelihood of remission.

Among 81 youth who continued on glucose-lowering medication, 84.0% (n = 68) continued on a single glucose-lowering agent, 37.0% (n = 30) were on 2 or more glucose-lowering agents, 22.2% (n = 18) were on insulin only, and 27.2% (n = 22) were on a glucose-lowering agent and insulin. At the last follow-up, HbA1c decreased, and weight and BMI increased among all Y-T2D regardless of remission status (Table 2). Youth who were in remission had the greatest change in HbA1c and were weight stable, but there were no significant differences between groups.

**Figure 2:**
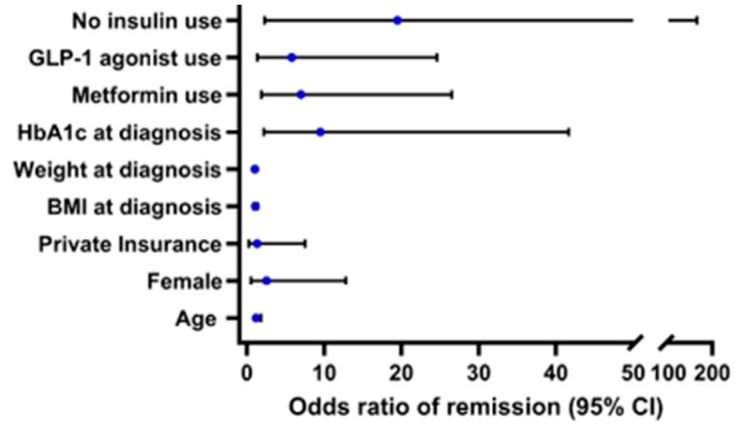
Odds ratios of remission (95% CI), Nagelkerke R^2^ = 0.782, *p* < 0.001. Reference groups for categorical variables are male for biologic sex and public insurance for insurance status.

Effects of covariates on achieving remission are described in Figure 2. Baseline medication use and HbA1c were related to remission, Nagelkerke R^2^ = 0.782, *p* < 0.001. Those with a lower HbA1c at the time of Y-T2D diagnosis were 9.5 times more likely to achieve remission (*p* = 0.003). There were no significant differences by remission status in age at diagnosis (*p* = 0.462), biologic sex (*p* = 0.522), race/ethnicity (NHB *p* = 0.999, Latinx *p* = 0.999), insurance type (*p* = 0.764), weight at diagnosis (*p* = 0.518), or BMI at diagnosis (*p* = 0.999).

## 4. Discussion

This follow-up study confirmed decreased Y-T2D incidence following the lifting of COVID-19 pandemic-related restrictions and demonstrated that low remission rates for youth diagnosed during the COVID-19 restrictions period remain. These findings highlight the significant impact of prolonged, multifaceted public health measures during the COVID-19 pandemic on Y-T2D incidence and the severity of presentation. As demonstrated in our previous study, the incidence and severity of Y-T2D increased markedly during the restriction period, with a notable decrease in the post-restriction period [1]. While virtual learning was a major visible change, it occurred alongside multiple concurrent interventions—including stay-at-home orders, suspension of organized sports, closure of recreational facilities, and reductions in preventive care—that likely acted synergistically to influence metabolic health. This study demonstrates a temporal association between COVID-19–related public health restrictions and both the incidence and severity of youth-onset T2D, rather than a proven causal relationship. Findings should therefore be interpreted descriptively. The pandemic restrictions functioned as a population-level social and behavioral disruption; although our design was descriptive, this framework highlights the importance of social context in shaping metabolic outcomes. The observed trends should also be viewed within a social and developmental framework, recognizing the pandemic as a disruption to multiple determinants of health, including schooling, physical activity, and stress.

The decline in incident cases after restrictions eased suggests that factors specific to the restrictions environment, rather than direct effects of COVID-19 infection, played a critical role in the observed trends [4]. Presenting the data within the framework of “public health restriction phases” rather than education modality alone improves interpretability and aligns more closely with the actual exposure experienced by youth. Rates of COVID-19 infection were low in our cohort (2.5% at diagnosis) and did not correlate with HbA1c at presentation [6]. Nevertheless, acute SARS-CoV-2 infection has been hypothesized to contribute to transient insulin resistance or β-cell dysfunction and may explain unusual glycemic presentations or more rapid disease progression in some youth. Emerging evidence suggests that targeted therapies addressing acute COVID-19-induced endocrine dysfunction—such as interventions that reduce systemic inflammation, preserve β-cell function, or attenuate insulin resistance—may offer a promising avenue to mitigate post-infectious dysglycemia and long-term metabolic complications in youth [16,17,18]. Incorporating such approaches into acute COVID-19 management could help reduce the risk of youth-onset diabetes following SARS-CoV-2 infection. In contrast, changes in lifestyle behaviors were pronounced during the initial stages of the COVID-19 pandemic. During the restrictions period, many youth experienced reduced physical activity, increased sedentary behavior, disruptions in dietary patterns, and heightened psychological stress, all of which have been associated with metabolic dysregulation and increased risk for T2D [19,20]. The lifting of public health restrictions likely facilitated improved lifestyle behaviors, social interactions, and access to healthcare, contributing to the observed reduction in new Y-T2D cases [21,22,23,24]. Comparable post-restriction patterns in youth-onset type 2 diabetes have also been observed internationally. Reports from pediatric diabetes networks in the United Kingdom, Canada, and Japan have documented increases in newly diagnosed Y-T2D during early pandemic restrictions, followed by partial normalization after the reopening of schools and clinics [25,26]. These data suggest that the temporal associations identified in our cohort reflect broader global behavioral and healthcare dynamics rather than phenomena unique to the United States. In addition to behavioral and environmental factors, the observed decline in Y-T2D incidence may also reflect broader public health efforts, including enhanced COVID-19 treatment strategies, reduced morbidity and mortality, and the protective effects of widespread vaccination campaigns during the post-pandemic period. However, our findings indicate that while incidence rates decreased, they did not return to pre-pandemic incidence (4.2 cases/month), and remission rates were relatively low, highlighting the long-term metabolic consequences of the pandemic-induced changes in lifestyle and healthcare access [2].

Notably, diabetes remission is a relatively new concept, and the term was coined in 2021 to standardize and systematically quantify glycemic response to more effective surgical and medical interventions for diabetes [14]. Yet, the definition of diabetes remission remains debatable, and data derived from adult studies provide inconclusive guidance. The applicability of this 3-month criterion to remission status for Y-T2D remains uncertain due to limited pediatric-specific research. Y-T2D often presents as more aggressive disease course compared to adult-onset T2D, with rapid β-cell decline and earlier onset of complications. The Treatment Options for Type 2 Diabetes in Adolescents and Youth (TODAY) study demonstrated that, despite initial glycemic control, many youths experienced loss of glycemic control over time, highlighting the challenges in this population [27]. Given these differences, some experts suggest that longer periods of normoglycemia may be necessary to confidently define remission in youths. However, specific guidelines or consensus statements addressing this issue in the pediatric population are currently lacking. Our current analysis provided novel insights into the characteristics of Y-T2D who achieved remission in a real-world setting. Lower HbA1c at the time of Y-T2D diagnosis was shown to be a significant predictor of achieving remission, suggesting that improved baseline beta-cell function since BMI, a surrogate for insulin sensitivity, was comparable across all youth. In adolescents, insulin resistance is known to transiently increase during puberty [28]. Some cases of Y-T2D may reflect a transient phenotype that remits as pubertal insulin sensitivity improves. Additionally, dysglycemia may have been transient in youth with mild COVID-related β-cell stress, raising the possibility of spontaneous resolution in some individuals. These findings align with previous data in the TODAY and RISE studies, confirming higher baseline beta-cell function as a predictor of good glycemic control [29,30]. Further, the use of glucose-lowering medications, particularly metformin and GLP-1 receptor agonists, was associated with a higher likelihood of remission [31]. In contrast, insulin therapy was linked to lower remission rates, which may reflect the severity of diabetes at diagnosis rather than a direct effect of the medication. These analyses support recommendations for early combination therapy with metformin and a GLP-1 receptor agonist to optimize first-line management in Y-T2D [32]. Greater odds for remission were also associated with social determinants of health. Lower rates were observed among underserved youth, emphasizing persistent disparities. Youth with public insurance had a lower likelihood of remission, emphasizing the importance of healthcare accessibility and support systems in diabetes management. NHB and Latinx youth, who were disproportionately affected by the pandemic-related surge in Y-T2D, continue to experience lower rates of remission, highlighting the need for targeted interventions that address social determinants of health [33]. Notably, though race and ethnicity were significantly associated with remission status in unadjusted analyses, this association was not sustained in the binomial logistic regression model. This discrepancy could be attributable to small sample sizes—particularly among Non-Hispanic White (NHW) participants, who served as the reference group. Our findings align with previous research indicating that multiple socio-environmental and ecological factors contribute significantly to the pathophysiology of Y-T2D. Observed racial and socioeconomic disparities reflect structural inequities in healthcare and community resources that were likely magnified during the pandemic. The prolonged metabolic burden imposed by public health restrictions underscored the importance of implementing early screening and preventive strategies in at-risk populations [34]. Future research should explore interventions aimed at mitigating the long-term impact of pandemic-related disruptions on youth metabolic health, including school-based health initiatives, community programs promoting physical activity, and policies ensuring equitable access to healthy foods and medical care.

This study provides further evidence of the detrimental effects of public health restrictions on incidence rates of youth-onset type 2 diabetes (Y-T2D) and highlights the challenges in achieving remission post-pandemic. Our results suggest education mode was a proxy for pandemic-associated environmental shifts rather than a causal determinant. While incidence declined after restrictions lifted, these patterns may reflect a new baseline of elevated risk rather than a full return to pre-pandemic conditions. Baseline demographic differences were small in magnitude, and statistical modeling confirmed that severity at presentation—not learning modality—was the key predictor of remission. While specific data on seasonal variations in Y-T2D incidence is limited, the SEARCH for Diabetes in Youth study found a significant increase in diagnoses occurring in August. The authors hypothesized that this may be due to an increase in physical exams prior to returning to school, which led to the detection of asymptomatic hyperglycemia [35]. Alternatively, it may be that during extended periods away from school, children engage in reduced physical activity and increased sedentary behavior, similar to the behaviors exhibited during the restriction period. Prioritizing structured activities like camps or community programs may help to maintain regular physical activity levels and mitigate these risks. Interpretations regarding behavioral contributors are speculative, as variables such as physical activity, diet, and stress were not systematically collected. Future interdisciplinary studies incorporating developmental stress theory and social determinants frameworks may enhance understanding of behavioral and contextual mediators of Y-T2D risk. Further research is needed to determine whether seasonal patterns exist in Y-T2D and if there is a correlation with reduced activity.

Our findings align with national and international pediatric diabetes trends, albeit in mixed-type cohorts. The TrenDY network in the U.S. reported that the frequency of youth-onset diabetes (including type 1 and type 2) increased sharply in the first year of the pandemic, driven largely by type 2 diagnoses [36]. Similarly, meta-analytic data from D’Souza et al. demonstrated elevated pediatric diabetes incidence during the COVID-19 period [37]. In parallel, acuity in diabetes presentation and DKA severity also increased among both T1D and T2D youth during the early pandemic, with subsequent reductions in severity post-pandemic [1,2,3,5]. While population-based registries such as SEARCH and DiabKids offer exemplary designs for causal insight, few currently publish T2D-specific results; the SWEET registry is among the few to document shifts in the proportion of T2 among pediatric diabetes cohorts [38]. Our single-center data contribute complementary longitudinal detail, underscoring the need for multicenter, type-specific studies integrating behavioral and socioeconomic dimensions. These population-based studies incorporate behavioral and socioeconomic variables that strengthen causal inference and contextual understanding. Our single-center design complements population study designs by providing granular, longitudinal data from a diverse metropolitan population, though future multicenter collaborations integrating behavioral, socioeconomic, and infection-related indicators will be critical to advance mechanistic insight.

While our study provides valuable insights into Y-T2D incidence and remission rates, several limitations should be acknowledged. This study is limited by its retrospective design and single-center population, which may introduce inaccuracies in medical records and missing data that may influence the analysis. Behavioral and socioeconomic variables were not uniformly available and may still contribute to the observed outcomes. Remission occurred in a small subset of patients, which may limit statistical precision and model stability. Behavioral variables (physical activity, diet, and stress) were not collected and therefore could not be directly analyzed. Because this was a retrospective, single-center chart review, causal inference is not possible. Our analysis compared discrete time periods rather than employing a counterfactual design, such as an interrupted time series. We therefore interpret findings descriptively rather than causally. Variability in local restrictions and healthcare access during the pandemic may also have influenced observed incidence trends. Approximately 46% of youth diagnosed during restrictions lacked sufficient longitudinal data, which may have introduced attrition bias, particularly as those lost may have had more favorable remission trajectories. COVID-19 status was not systematically recorded. Multivariable adjustment for socioeconomic and racial confounding was not performed. Diagnostic misclassification is possible where antibody data were incomplete. Approximately half of restriction-period cases had sufficient follow-up, and attrition could bias remission estimates toward lower rates. Temporal boundaries were defined to align with the regional pattern of school closures and policy mandates but should be viewed as approximations of social exposure periods, recognizing variation across districts. As a tertiary pediatric referral center serving a predominantly urban and publicly insured population, our findings may not generalize to non-urban or privately insured community settings and populations. Future studies should adopt cross-disciplinary approaches integrating medical, behavioral, and policy data, such as school reopening metrics, physical activity tracking, and socioeconomic indicators. Linking such data will clarify mechanisms underlying observed temporal patterns.

## 5. Conclusions

In summary, while incidence rates declined following the lifting of pandemic-related restrictions and the return to in-person learning, remission rates remained low, particularly among racial and socioeconomic minority groups. Early glycemic control and targeted therapeutic interventions appear to improve remission rates, emphasizing the need for proactive diabetes management strategies. These findings underscore the importance of considering pediatric metabolic health when designing and implementing prolonged public health restrictions in future emergencies. Given the potential long-term health consequences of pandemic-related metabolic disruptions, continued research and public health efforts are essential to support at-risk youth and reduce disparities in diabetes outcomes. These findings demonstrate temporal associations, not causal effects, and emphasize the importance of ongoing surveillance and equitable access to care. This descriptive study contributes longitudinal data on youth-onset type 2 diabetes trends before, during, and after pandemic-related restrictions, providing context for future mechanistic and population-based studies. These findings should inform future educational and healthcare policies to mitigate the adverse effects of virtual learning on pediatric metabolic health.

## Figures and Tables

**Table 1:** Demographic and clinical characteristics of newly diagnosed youth-onset type 2 diabetes.

	Pandemic Restrictions Period (n = 235) *	Post-Restrictions Period (n = 154) *	Mean Difference (95% CI)	*p*-Value	Effect Size
Cases Diagnosed per 30 days	13.2	6.3	-	-	-
Female Sex, n (%)	107 (45.5%)	83 (53.9%)	+0.08 (−0.02, 0.19)	0.107	-
Age at Diagnosis, mean (SD)	14.5 (2.2)	14.8 (2.5)	+0.30 (−0.18, 0.78)	0.213	g = 0.12
Race/Ethnicity, n (%)					
NHW	10 (4.3%)	12 (7.8%)	+0.04 (−0.02, 0.09)	0.095	-
NHB	173 (73.6%)	80 (51.9%)	−0.22 (−0.31, −0.12)	**<0.001**	v = 0.24
Latinx	44 (18.7%)	46 (29.9%)	+0.11 (0.00, 0.22)	**0.003**	v = 0.18
Other	8 (3.4%)	16 (10.4%)	+0.07 (0.02, 0.12)	**0.005**	v = 0.07
Insurance, n (%)					
Private	38 (16.2%)	46 (29.9%)	+0.14 (0.05, 0.22)	**0.001**	v = 0.16
Public	197 (83.8%)	100 (64.9%)	−0.19 (−0.28, −0.10)	**<0.001**	v = 0.22
Other	0 (0%)	8 (5.2%)	-	-	v = 0.10
In-patient Management, n (%)	136 (57.9%)	55 (35.7%)	−0.22 (−0.32, −0.13)	**<0.001**	v = 0.21
Hemoglobin A1c					
(%), mean (SD)	10.1 (2.3)	9.1 (2.5)	−1.00 (−1.49, −0.51)	**<0.001**	g = 0.42
(mmol/mol), mean (SD)	85.4 (27.8)	76.3 (27.3)	−9.10 (−15.50, −2.70)	**0.002**	
Random Blood Glucose					
(mg/dL), median (IQR)	308 (IQR 187–452)	219 (IQR 122–350)	−89.00 (−125.00, −53.00)	**<0.001**	g = 0.38
(mmol/L), median (IQR)	16.2 (IQR 7.7–23.6)	11.7 (IQR 6.4–19.3)	−4.50 (−5.95, −3.05)	**<0.001**	
Venous pH, median (IQR)	7.35 (IQR 7.22–7.38)	7.35 (IQR 7.32–7.40)	0.00 (−0.02, 0.02)	**0.049**	g = 0.15
Serum Bicarbonate (mmol/L), median (IQR)	23 (IQR 16–26)	25 (IQR 22–27)	+2.00 (0.89, 3.11)	**<0.001**	g = 0.24
Diabetic Ketoacidosis, n (%)	49 (20.9%)	11 (7.1%)	−0.14 (−0.20, −0.07)	**<0.001**	
Diabetic Ketoacidosis Severity, n (%)					
Mild	18 (7.7%)	4(2.6%)	−0.05 (−0.09, −0.01)	**0.035**	v = 0.12
Moderate	11 (4.7%)	3 (1.9%)	−0.03 (−0.06, 0.01)	0.157	v = 0.09
Severe	20 (8.5%)	4 (2.6%)	−0.06 (−0.10, 0.02)	0.178	v = 13
Hyperosmolar Diabetic Ketoacidosis, n (%)	15 (6.4%)	1 (0.6%)	−0.06 (−0.10, 0.02)	**0.005**	
Hyperglycemic Hyperosmolar State, n (%)	0 (0%)	2 (1.3%)	+0.01 (−0.01, +0.03)	0.157	
Weight, kg					
mean (SD)	105 (25)	102 (29)	−3.00 (−8.59, 2.59)	0.278	
Z-score (SD)	2.72 (0.65)	2.46 (0.88)	−0.26 (−0.42, −0.10)	0.078	
BMI, kg/m^2^					
mean (SD)	36.5 (7.0)	37.1 (9.2)	+0.60 (−1.11, 2.31)	0.467	g = 0.07
Z-score (SD)	2.38 (0.40)	2.31 (0.52)	−0.07 (−0.17, 0.03)	0.135	g = 0.09
Obesity Class, n (%)					
Overweight	8 (3.4%)	15 (9.7%)	+0.06 (0.01, 0.12)	**0.019**	v = 0.16
Class 1	43 (18.3)	29 (18.8%)	+0.01 (−0.07, 0.08)	0.751	v = 0.01
Class 2	74 (31.5%)	39 (25.3%)	−0.06 (−0.15, 0.03)	0.053	v = 0.12
Class 3	80 (34.0%)	65 (42.2%)	+0.08 (−0.02, 0.18)	0.356	v = 0.08

* Totals may not sum due to missing data or individuals categorized as “Other”. Percentages may exceed 100% due to rounding. Effect sizes included: Cramer’s V (categorical) and Hedges’ g (continuous); <0.20 considered small.

**Table 2:** Demographic Characteristics of Y-T2D Youth Diagnosed during Public Health Restrictions grouped by Remission Status after 2 years of follow-up.

	Remission (n = 26)	Non-Remission, Medications (n = 81)	Non-Remission, No Medications (n = 19)	*p*-Value
Female Sex, n (%)	10 (38.5)	40 (49.4)	9 (47.4)	0.623
Age at Diagnosis, mean (95% CI)	14.5 (13.8, 15.2)	13.8 (13.3, 14.3)	13.0 (12.3, 13.6)	**0.031**
T2D Duration, months, mean (95% CI)	12.4 (10.5, 14.4)	12.9 (10.7, 15.1)	15.5 (12.1, 18.9)	0.481
Race/Ethnicity, n (%)				**0.047**
NHW	0 (0.0)	4 (4.9)	4 (21.1)	-
NHB	19 (73.1)	62 (76.5)	12 (63.2)	0.488
Latinx	7 (26.9)	15 (18.5)	3 (15.8)	0.575
Private Insurance, n (%)	21 (80.8)	71 (87.8)	16 (84.2)	0.669
Hemoglobin A1c, mean (95% CI) %	9.6 (8.0, 11.2)	10.1 (9.6, 10.6)	9.4 (8.4, 10.4)	0.626
mmol/mol	81 (73.0, 89.0)	87 (86.6, 87.4)	79 (78.5, 79.5)	
Weight, kg, mean (95%CI)	108.7 (99.2, 118.3)	106.5 (101.2, 111.9)	101.3 (90.0, 112.7)	0.586
BMI, kg/m^2^, mean (95%CI)	37.0 (33.9, 40.1)	37.2 (35.6, 38.7)	36.7 (32.5, 40.8)	0.970
Glucose Lowering Medications, n (%)				
Metformin/Metformin ER	4 (15.4)	39 (48.1)	8 (42.1)	**0.012**
GLP-1	3 (11.5)	31 (38.3)	5 (26.3)	**0.033**
SGLT2	0 (0.0)	0 (0.0)	0 (0.0)	-
TZD	0 (0.0)	1 (1.2)	0 (0.0)	0.756
Insulin	1 (3.8)	38 (46.9)	1 (5.3)	**<0.001**
Delta Hb A1c (%) *	−4.2 ± 3.8	−2.6 ± 3.0	−2.1 ± 2.8	0.086
Delta weight (kg) *	0.81 ± 13.86	8.47 ± 13.80	7.78 ± 10.33	0.065
Delta BMI (kg/m^2^) *	2.04 ± 12.22	3.53 ± 17.87	2.67 ± 2.61	0.932

* Last clinic follow-up—baseline. Data are mean (95% CI) or n (%).

## Data Availability

All data generated or analyzed during this study are included in this article. Further inquiries can be directed to the corresponding author.

## Abbreviations

The following abbreviations are used in this manuscript:

ADA	American Diabetes Association
BMI	Body Mass Index
BUN	Blood Urea Nitrogen
CI	Confidence Interval
COVID-19	Coronavirus Disease 2019
DKA	Diabetic Ketoacidosis
EMR	Electronic Medical Record
GAD65	Glutamic Acid Decarboxylase 65 antibody
GLP-1	Glucagon-Like Peptide-1
HbA1c	Hemoglobin A1c
HHS	Hyperglycemic Hyperosmolar Syndrome
IA2	Islet Antigen-2 Antibody
ISPAD	International Society for Pediatric and Adolescent Diabetes
IQR	Interquartile Range
NHB	Non-Hispanic Black
NHW	Non-Hispanic White
OR	Odds Ratio
RISE	Restoring Insulin Secretion Study
SD	Standard Deviation
SGLT2	Sodium-Glucose Cotransporter-2 inhibitor
SPSS	Statistical Package for the Social Sciences
T2D	Type 2 Diabetes
TODAY	Treatment Options for Type 2 Diabetes in Adolescents and Youth
TZD	Thiazolidinedione
Y-T2D	Youth-Onset Type 2 Diabetes
ZNT8	Zinc Transporter 8 antibody
